# Fluorescence and Docking Studies of the Interaction between Human Serum Albumin and Pheophytin

**DOI:** 10.3390/molecules201019526

**Published:** 2015-10-27

**Authors:** Otávio Augusto Chaves, Ana Paula de O. Amorim, Larissa H. E. Castro, Carlos Mauricio R. Sant’Anna, Márcia C. C. de Oliveira, Dari Cesarin-Sobrinho, José Carlos Netto-Ferreira, Aurélio B. B. Ferreira

**Affiliations:** 1 Departamento de Química, Universidade Federal Rural do Rio de Janeiro, BR 465, km 47, 23890-000 Seropédica-RJ, Brazil; E-Mails: otavio_ufrrj@hotmail.com (O.A.C.); anamorim16@gmail.com (A.P.O.A.); larissa_hec@hotmail.com (L.H.E.C.); santanna@ufrrj.br (C.M.R.S.A.); mccdeo@gmail.com (M.C.C.O.); dari@ufrrj.br (D.C.-S.); jcnetto.ufrrj@gmail.com (J.C.N.-F.); 2 Instituto Nacional de Metrologia, Qualidade e Tecnologia-INMETRO, Divisão de Metrologia Química, 25250-020 Duque de Caxias-RJ, Brazil

**Keywords:** *Talinum triangulare*, pheophytin, human serum albumin, fluorescence spectroscopy, docking

## Abstract

In the North of Brazil (Pará and Amazonas states) the leaves of the plant *Talinum triangulare* (popular: *cariru*) replace spinach as food. From a phytochemical point of view, they are rich in compounds of the group of pheophytins. These substances, related to chlorophyll, have photophysical properties that give them potential application in photodynamic therapy. Human serum albumin (HSA) is one of the main endogenous vehicles for biodistribution of molecules by blood plasma. Association constants and thermodynamic parameters for the interaction of HSA with pheophytin from *Talinum triangulare* were studied by UV-Vis absorption, fluorescence techniques, and molecular modeling (docking). Fluorescence quenching of the HSA’s internal fluorophore (tryptophan) at temperatures 296 K, 303 K, and 310 K, resulted in values for the association constants of the order of 10^4^ L∙mol^−1^, indicating a moderate interaction between the compound and the albumin. The negative values of Δ*G*° indicate a spontaneous process; Δ*H*° = 15.5 kJ∙mol^−1^ indicates an endothermic process of association and Δ*S*° = 0.145 kJ∙mol^−1^∙K^−1^ shows that the interaction between HSA and pheophytin occurs mainly by hydrophobic factors. The observed Trp fluorescence quenching is static: there is initial non-fluorescent association, in the ground state, HSA:Pheophytin. Possible solution obtained by a molecular docking study suggests that pheophytin is able to interact with HSA by means of hydrogen bonds with three lysine and one arginine residues, whereas the phytyl group is inserted in a hydrophobic pocket, close to Trp-214.

## 1. Introduction

The *Portulacaceae* family consists of annual fleshy herbs, with approximately 30 genera and about five hundred species, predominantly from tropical and subtropical regions of Africa and the Americas, but some species can occasionally be found in Europe, Asia and Oceania [1,2]. The *Talinum* genus belongs to the Portulacaceae family, and includes about fifty species distributed in the tropics, subtropics and temperate regions of the world; one of these species, *Talinum* *triangulare* (syn, *Talinum fruticosum auct. non* (L.) *Juss*.), popularly known in Brazil as joão gomes, *língua de vaca*, *major gomes*, *manjogomes*, *caruru do Pará* and *cariru* [3], is an unconventional vegetable foodstuff, consumed mainly in the northern region, especially in Amazonas and Pará states, due to its high nutritional content and can be used in child nutrition as a spinach (*Spinacia oleracea*) substitute [1].

Reports in the scientific literature show that *Talinum triangulare* has antioxidant activities and the hydromethanolic extract of the stalk is a source of allantoin, aspartic acid, mixed steroidal saponins, and special metabolites as acrylamides, among other nitrogen compounds. The dichloromethane-ethyl acetate extract of the leaves is rich in compounds of the pheophytin class of special metabolites, analogues of porphyrins, which have the ability to form metal complexes [4,5].

Recent studies have shown that the hydroethanolic extract of the leaves of *Talinum triangulare* has antitumor activity on leukemic lines HL-20, K-562, Lucena, Jukart and lung cancer H-460 [5]. In addition, stimulating activity of brain function in laboratory mice is attributed to ingestion of the plant [6].

Albumin is a protein of high biological importance. It is present in egg white, milk, and blood, where it is very abundant in the plasma (serum albumin) and is the main protein. Many researchers have studied the structure and properties of serum albumin and its interactions with other proteins and ligands in order to understand its functions in the body [7].

Serum albumins of the circulatory system have various physiological functions, including the maintenance of osmotic pressure, transport, distribution, and participation in the metabolism of many endogenous and exogenous ligands (e.g., drugs, metabolites, fatty acids, amino acids, and hormones), resulting in increased solubility of these compounds in the plasma, which can reduce their toxicity, and/or protect them against oxidation or other reactions [8,9]. The binding between drugs and serum albumin is an important factor in understanding the interaction of the organism with drugs (pharmacokinetic studies), since it influences the distribution, excretion, metabolism and interaction with the biological target, itself.

**Figure 1:**
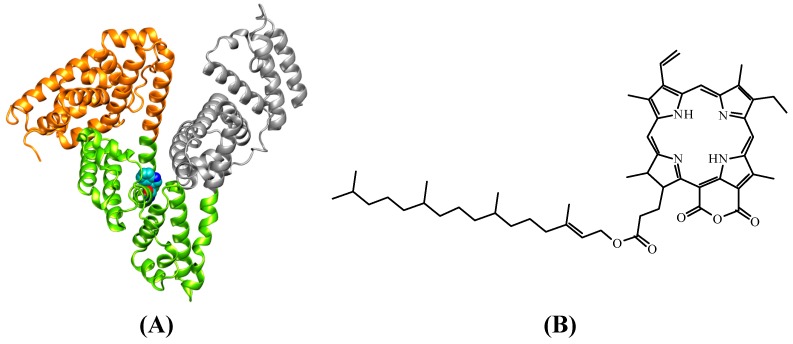
(**A**) Crystallographic structure of HSA (PDB: 1N5U), showing each of its domains: I (Brown), II (green), and III (gray). Trp214 residue, located in domain II, is presented in the space-filling mode (green); (**B**) Structural formula of pheophytin (17*R*,18*R*)-purpurin 18-phytyl ester (18-FP).

HSA structure consists of three structurally-similar domains (I, II, and III), each containing two subdomains, A and B [10,11] (Figure 1A). Each subdomain has a main cavity for interaction with ligands and, therefore, there are a total of six main cavities for interaction. 

The amino acid residue tryptophan (Trp) is often used for the association studies of this albumin with endogenous and exogenous molecules by fluorescence spectroscopy techniques. The HSA structure has only one Trp, located in subdomain IIA (Trp-214), as shown in Figure 1 [12].

The presence of molecules that interact with HSA can modify the fluorescence of HSA and this effect depends on the concentration and average distance between these molecules and the indole moiety of the Trp chromophore. What typically occurs is a decrease in fluorescence intensity and, therefore, it is said that the molecule is a quencher of the fluorescence.

## 2. Results and Discussion

### 2.1. Molecular Absorption Spectroscopy in the UV-Vis

Proteins typically have an absorption band at 280 nm corresponding to the absorption of UV light for three types of aromatic residues: tryptophan (Trp), phenylalanine (Phe), and tyrosine (Tyr). Among the three aromatic amino acids mentioned, the most intense absorption and emission is that of Trp, which has higher molar absorptivity and intrinsic fluorescence quantum yield than both tyrosine and phenylalanine [13].

**Figure 2:**
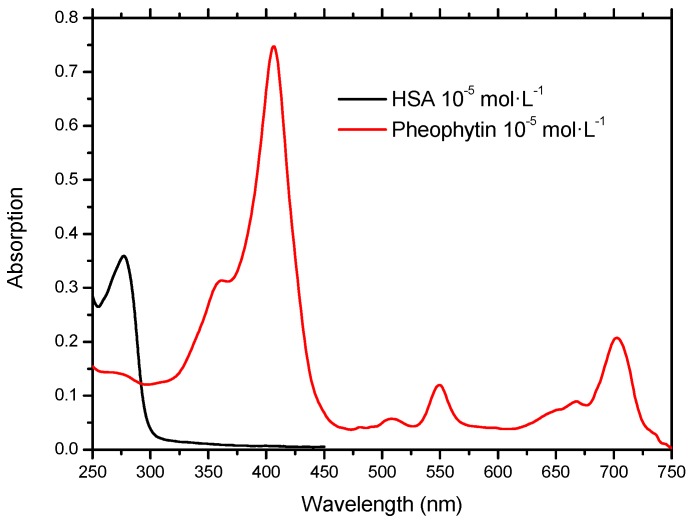
Absorption spectra UV-Vis of the HSA solution in pH = 7.4 and pheophytin in ethanol.

The treatment of certain types of cancers by photodynamic therapy, can use pheophytins—organic molecules classified as second-generation photosensitizers [14]. The pheophytin (17*R*,18*R*)-purpurin 18-phytyl ester (18-FP) (Figure 1B)—isolated from the plant *Talinum triangulare*—besides absorbing at 400 nm, due to absorption by the Soret band [15], also absorbs at longer wavelengths, 550 nm and 700 nm. The discovery and development of molecules which absorb radiation in the near infrared (600–900 nm) should provide new opportunities for contrast agents for medical imaging and photodynamic therapy. This is due to the high penetration depth of this radiation in the tissues, without causing health damage [16]. Figure 2 shows the absorption spectra of HSA and pheophytin solutions.

### 2.2. Fluorescence Spectroscopy

Fluorescence quenching of a substance by interaction with another, which is added in increasing amounts, can be used as a technique to measure the binding affinities between some macromolecules and ligands acting as suppressors (quenchers). This quenching is expressed as the decrease in the quantum yield of the fluorophore fluorescence induced by a variety of molecular interactions [13].

**Figure 3:**
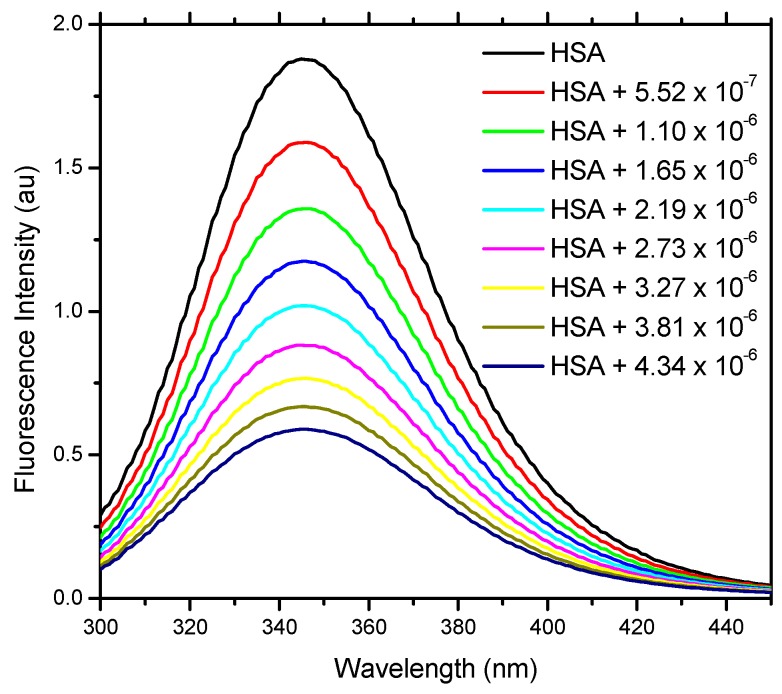
Fluorescence emission spectra of the internal tryptophan residue of HSA and its fluorescence quenching by the addition of 18-FP. HSA in PBS buffer solution (pH 7.4), C_HSA_ = 1.00 × 10^−5^ mol∙L^−1^, T = 296K, λ_exc_ = 280 nm.

Figure 3 shows the quenching of the fluorescence emission of the Trp residue of HSA by addition of aliquots of 18-FP solution. This indicates that the location of the pheophytin molecule inside the protein should be close to the Trp residue [17]. The absence of considerable changes in the wavelength of emission maximum of HSA is evidence that the presence of pheophytin does not exert a great influence on the polarity of the microenvironment of the cavity around the Trp residue [18].

#### 2.2.1. Binding Constant of Stern-Volmer Modified (*K*_a_)

**Figure 4:**
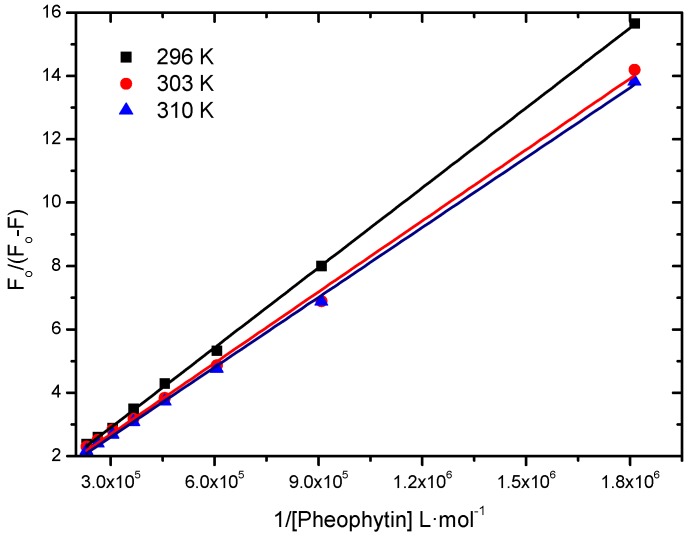
Modified Stern-Volmer plots of the fluorescence quenching of the HSA by pheophytin 18-FP at various temperatures.

To analyze if the interaction between HSA and the pheophytin 18-FP is strong, moderate, or weak, we calculated the modified Stern-Volmer binding constant (*K*_a_) [19,20]. This constant is obtained according to Equation (1) (Figure 4 and Table 1):

(1) $$\frac{F_{0}}{F_{0}-F}=\frac{1}{fK_{a}}\frac{1}{\left[Q\right]}+\frac{1}{f}$$

where *F*_0_ and *F* are the fluorescence intensities of HSA without and with the quencher (pheophytin) at 350 nm, respectively; *K*_a_ is the modified Stern-Volmer binding constant; ƒ the fraction of the initial fluorescence that is accessible to the quencher and [*Q*], the quencher concentration.

**Table 1:** *K*_a_ values at 296 K, 303 K, and 310 K.

*T* (K)	*K*_a_ (L∙mol^−1^)	*r*^2^
296	4.48 ± 0.06 × 10^4^	0.9997
303	5.43 ± 0.13 × 10^4^	0.9978
310	5.95 ± 0.08 × 10^4^	0.9992

The *K*_a_ values are of the order of 10^4^ L∙mol^−1^, indicating a moderate interaction between albumin and pheophytin and that there is an initial association HSA:pheophytin [18,21,22]. Therefore, this pheophytin can be carried by albumin. Moderate association is preferable over strong or weak association (high or low *K*_a_ values), because the pheophytin could be irreversibly retained or not carried in these cases, respectively. Although there was an increase in binding constant with increasing temperature, this increase does not seem to be significant. The binding constant values depend on the charge and structure of the pheophytin, as well as the conformation and charge of HSA [23].

#### 2.2.2. Thermodynamics Parameters (Δ*G*°, Δ*H*°, Δ*S*°)

**Figure 5:**
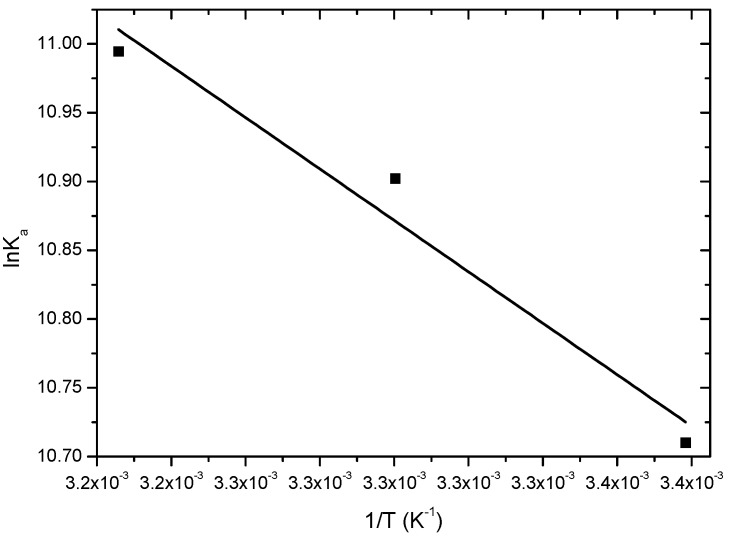
Van’t Hoff graph of *K*_a_ values from modified Stern-Volmer plots at 296 K, 303 K, and 310 K.

To get some insight on the thermodynamic parameters Δ*G*°, Δ*H*°, Δ*S*° controlling the interaction BSA:18-FP, data from Table 1 were plotted following the van’t Hoff Equation (2A) (Figure 5) and Δ*G*° was calculated from the Gibbs free energy Equation (2B) [24] (Table 2).

(2A) $$\text{ln}K_{a}=\frac{\Delta H^{0}}{RT}+\frac{\Delta S^{0}}{R}$$

(2B) $$\Delta G^{0}=\Delta H^{0}-T\Delta S^{0}$$

where Δ*H*°, Δ*S*°, and Δ*G*° are the enthalpy, entropy, and Gibbs free energy, respectively, for the HSA:18-FP association; *R* is the gas constant (*R* = 8.314 × 10^−3^ kJ/mol∙K), *T* are the temperatures (296 K, 303 K and 310 K) and *K*_a_ the binding constant.

**Table 2:** Thermodynamic parameters of interaction between HSA and 18-FP pheophytin.

*T* (K)	Δ*H*° (kJ∙mol^−1^)	Δ*S*° (kJ∙mol^−1^∙K^−1^)	Δ*G*° (kJ∙mol^−1^)	*r*^2^
296			−26.4	
303	15.5 ± 1.2	0.145	−27.4	0.9313
310			−28.4	

The negative value of Δ*G*° is consistent with spontaneous binding, the positive value of Δ*H*° indicates that the binding process of the pheophytin is endothermic, and the positive value of Δ*S*° shows that the interaction is mainly due to hydrophobic factors [25,26].

These hydrophobic factors are related to the influence of hydration molecules. There are two possible contributions that may explain the increase in entropy: hydration molecules are expelled with the entry of 18-FP into the protein cavity; desolvation of the pheophytin as it enters the cavity causes an increase in the number of micro-states of the system.

#### 2.2.3. Fluorescence Quenching; Static *vs.* Dynamic

**Figure 6:**
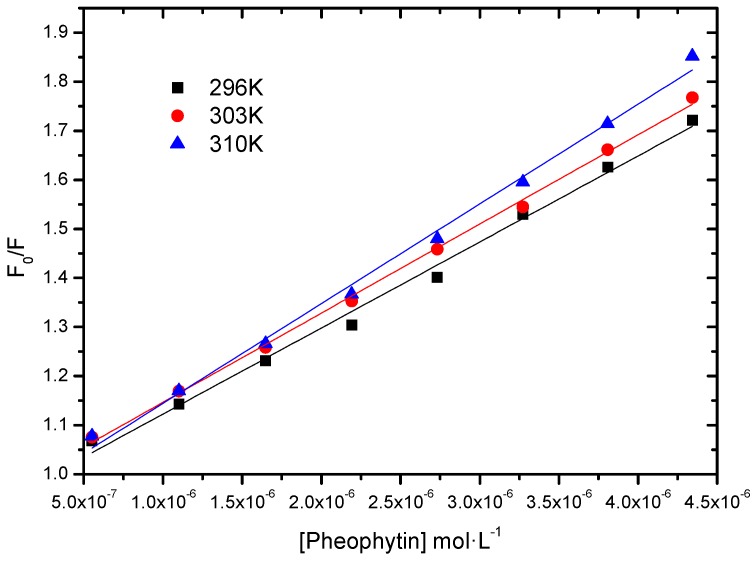
Stern-Volmer plot of fluorescence suppression of HSA by pheophytin at different temperatures.

Applying Equation (3A) (Figure 6) and Equation (3B) to each of the three temperature values, we can obtain the Stern-Volmer quenching constant (*K_SV_*) and the quenching rate constant (*k_q_*), respectively. These constants offer indications if the probable process of quenching of the internal Trp fluorescence of HSA is static or dynamic [20]. *K_SV_* and *k_q_* values are shown in Table 3.

(3A) $$\frac{F_{0}}{F}=1+k_{q}\tau_{0}\left[Q\right]=1+K_{SV}\left[Q\right]$$

(3B) $$k_{q}=\frac{K_{SV}}{\tau_{0}}$$

where *F*_0_ and *F* are the fluorescence intensities of HSA without and with the quencher (18-FP pheophytin), respectively; *K_SV_* is the Stern-Volmer quenching constant, *k_q_* is the quenching rate constant of HSA fluorescence, [Q] the quencher concentration, and τ_0_ is the lifetime of HSA in the absence of quencher (10^−8^ s) [18,27].

**Table 3:** *K_SV_* and *k_q_* values at 296 K, 303 K and 310 K.

*T* (K)	*K_SV_* (L∙mol^−1^)	*k_q_* (L∙mol^−1^∙s^−1^)	*r*^2^
296	1.75 ± 0.07 × 10^5^	1.75 × 10^13^	0.9927
303	1.82 ± 0.03 × 10^5^	1.82 × 10^13^	0.9980
310	2.03 ± 0.06 × 10^5^	2.03 × 10^13^	0.9946

Both static and dynamic quenching requires contact between fluorophore and quencher. In the first case, there is formation of a pre-complex and in the second, quenching occurs during collision, followed by separation. The diffusion limited rate constant (*k_d_* ~ 5 × 10^9^ L∙mol^−1^∙s^−1^ in water at 298 K), has a smaller value, compared to the values of the quenching rate constants (*k_q_* > 10^13^ L∙mol^−1^∙s^−1^), indicating that the probable mechanism of fluorescence quenching of HSA by 18-FP is static [18,20,28]; there is initial non-fluorescent association, in the ground state, between the fluorophore and the quencher.

### 2.3. Molecular Modeling Calculation

The three-dimensional structure of albumin has different binding sites with different specificities (domains I, II, and III); domains I (Sudlow I) and II (Sudlow II) are the most important [29]. Site I, also named warfarin binding site, is located in the IIA subdomain, while the binding site II, named indole/benzodiazepine binding site, is located in subdomain IIIA. Human serum albumin (HSA) is composed of a single polypeptide chain of 585 amino acid residues [30] and contains only one Trp residue, Trp-214, located in subdomain IIA. Trp-214 is located at an internal site of the protein with high hydrophobic character [31,32].

From the studies of HSA fluorescence quenching, it is known that the interacting pheophytin is located next to the Trp-214 amino acid residue. Studies using molecular modeling were performed to analyze the main intermolecular interactions between the pheophytin and the amino acid residues present in the subdomain IIA interaction cavity. In agreement with the experimental results, we suggest from the docking results that, even pheophytin being a fairly bulky molecule, it is able to be accommodated in the cavity next to the Trp-214 residue presenting a favorable interaction profile with the cavity residues.

**Figure 7:**
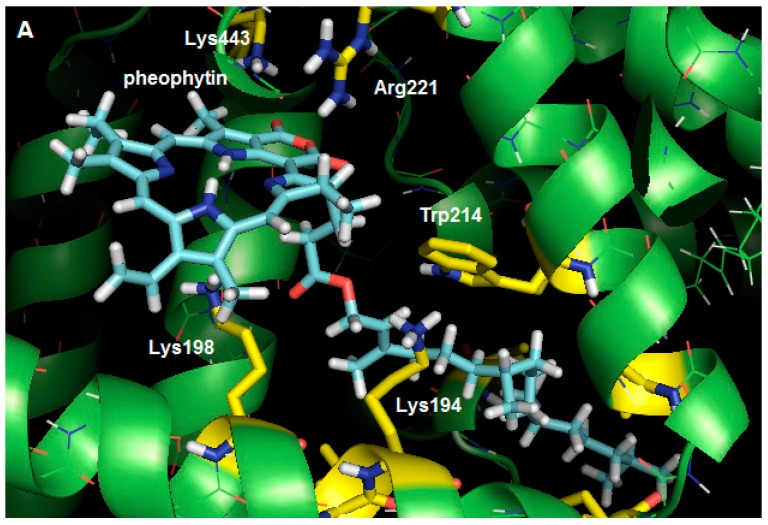

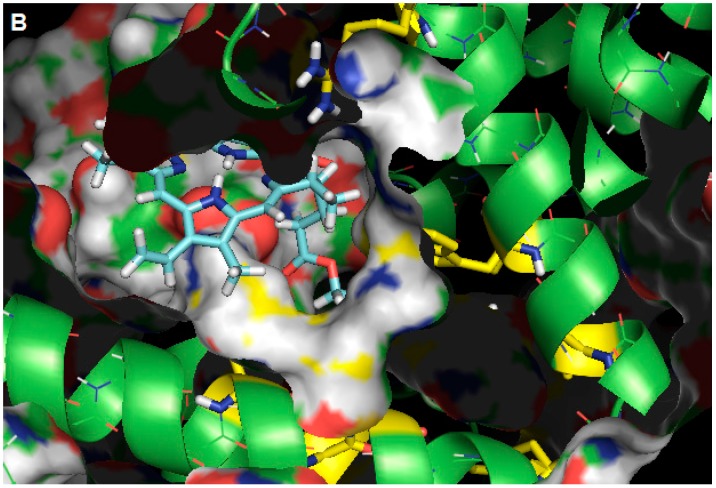
(**A**) Best score pose for pheophytin in HSA, obtained by molecular docking (*ChemPLP* function). Carbon: cyan (pheophytin), green (HSA), yellow (selected residues); hydrogen: white; oxygen: red; and nitrogen: blue; (**B**) Representation of the molecular surface of HSA, where it can be seen that the apolar side chain of pheophytin is completely surrounded by the hydrophobic gorge (figures generated with the PyMOL software).

Within the interaction cavity pheophytin interacts via hydrogen bonds with four amino acid residues—one arginine and three lysine residues (Figure 7). The Lys-198 and Lys-194 residues make hydrogen bonds with both oxygens of the phytyl ester group, establishing distances between the donor and acceptor atoms in the hydrogen bonds of 3.26 Å and 3.06 Å, respectively. The carboxyl groups of the cyclic anhydride receive hydrogen bonds from the Arg-221 and Lys-443 residues, with a distances of 3.26 Å and 2.57 Å, respectively. In addition to these interactions, the ligand appears to interact with HSA through a large number of hydrophobic interactions. The molecular docking results show that phytyl, a non-polar group, is accommodated in an essentially hydrophobic gorge within the HSA, where it interacts primarily with Leu-197, Phe-205, Ala-209, Leu-346, and Val-481 residues, in addition to the residue which is responsible for the fluorescence, Trp-214. This proximity between the quencher and the fluorophore can explain the efficiency of the fluorescence quenching [13].

## 3. Experimental Section 

### 3.1. Spectroscopic Experiments

#### 3.1.1. Instruments and Materials

Absorption spectra in the ultraviolet-visible region (UV-Vis) were obtained in Shimadzu model Mine 1240 (Kyoto, Japan), with an optical path quartz cell of 1 cm. Fluorescence spectra were performed on a Jasco Model J-815 spectrofluorometer (Easton, MD, USA) with a quartz cell of 1 cm optical step thermostated system equipped with Jasco PFD-425S15F 0.1 °C accuracy. 

Human Serum Albumin and sachet of phosphate buffer pH = 7.4 are both commercial products acquired from Aldrich Company. Ethanol, spectrophotometric grade, was acquired from Vetec (Rio de Janeiro-RJ, Brazil). The pheophytin (17*R*,18*R*)-purpurin 18-phytyl ester (18-FP, Figure 1B) was obtained from the Natural Products Chemistry Group of the Chemistry Department of UFRRJ (Seropédica-RJ, Brazil) [33].

#### 3.1.2. Methodology for UV-Vis Spectroscopy

Absorption spectra in UV-Vis were recorded in the 200–800 nm range for HSA solutions (1.00 × 10^−5^ mol∙L^−1^) in PBS buffer (pH = 7.4), and for the pheophytin (17*R*,18*R*)-purpurin 18-phytyl ester (18-FP) (1 × 10^−5^ mol∙L^−1^) in ethanolic solution.

#### 3.1.3. Methodology for Fluorescence Spectroscopy

Fluorescence emission spectra of HSA were recorded (λ_ex_ = 280 nm) for 1.00 × 10^−5^ mol∙L^−1^ solution (PBS buffer, pH = 7.4) in the range 300–450 nm, at temperatures 296 K, 303 K, and 310 K.

Initially the fluorescence emission spectra of an aliquot of 3 mL of albumin solution (1.00 × 10^−5^ mol∙L^−1^) was measured: this gave, at the emission maximum (λ_max_ of Trp-214 is 345 nm), the initial fluorescence intensity (*F*_0_). Then, volumes of pheophytin quencher 1.00 × 10^−3^ mol∙L^−1^ stock solution were added, so as to obtain 0.55, 1.10, 1.65, 2.19, 2.73, 3.27, 3.81, and 4.34 × 10^−6^ mol∙L^−1^, respectively, final concentrations; the variation of fluorescence emission intensity (*F*) with the quencher concentration [*Q*] was measured. Applying the modified Stern-Volmer, van’t Hoff, and Gibbs free energy equations [13], one can determine the association constant between biological macromolecules and ligands (*K*_a_), Stern-Volmer quenching constant (*K_SV_*), quenching rate constant (*k_q_*), and thermodynamic parameters (Δ*G*°, Δ*H*° and Δ*S*°).

### 3.2. Computational Experiments

#### Methodology for Docking Study

The crystallographic structure of human serum albumin was obtained from the Protein Data Bank (PDB) with access code is 1N5U [34]. This structure has a resolution of 1.90 Å. The structure of pheophytin (17*R*,18*R*)-purpurin 18-phytyl ester (18-FP) was built and energy-minimized with the semi-empirical method AM1 [35], available in the Spartan’14 program (Wavefunction, Inc., Irvine, CA, USA).

The docking was performed with the Gold 5.2 program (Cambridge Crystallographic Data Centre, Cambrige, CB2 1EZ, UK). Hydrogen atoms were added to the protein according to the ionization and tautomeric states inferred by the program [36].

Docking interaction cavities in the protein were established with radius of 10 Å and 15 Å from Trp-214. Better results were obtained with radius of 10 Å. The number of genetic operations (crossover, migration, mutation) in each docking run used in the searching procedure was set to 100,000. The program optimizes hydrogen-bond geometries by rotating hydroxyl and amino groups of amino acid side chains. The scoring function used was “ChemPLP” [37], which is the default function of the GOLD program. The score of each pose identified is calculated as the negative of the sum of a series of energy terms involved in the protein-ligand interaction process, so that the more positive the score, the better is the interaction. Figures of the molecular docking results were generated by PyMOL 1.1eval (Delano Scientific LLC program, Palo Alto, CA, USA).

## 4. Conclusions

Compared with other porphyrins and pheophytins from the literature, the pheophytin (17*R*,18*R*)-purpurin 18-phytyl ester (18-FP) binds moderately to human serum albumin. The association constant value (*K*_a_ ≈ 10^4^ L∙mol^−1^) is favorable for its efficient biodistribution by blood plasma. The thermodynamic parameters suggest that the spontaneous interaction (Δ*G*° < 0) is endothermic (Δ*H*° > 0), being entropically-driven (Δ*S*° > 0), and taking place mainly by hydrophobic factors due to the effect of hydration molecules (it is possible that hydration molecules are expelled with the entry of 18-FP into the protein cavity, and/or desolvation of the pheophytin as it enters the cavity causes an increase in the number of micro-states of the system). The quenching rate constant (*k_q_*) values in the order of 10^13^ L∙mol^−1^∙s^−1^ indicate that the process of Trp fluorescence quenching is static, with the non-fluorescent association HSA: pheophytin.

The docking results suggest that pheophytin interacts by means of hydrogen bonds with three lysine and one arginine residues. The ligand also interacts with HSA by means of its apolar side chain, which is inserted in a gorge surrounded by the side chains of hydrophobic amino acids (Leu-197, Phe-205, Ala-209, Leu-346, and Val-481 residues), including the fluorophore Trp-214, which could be related to the entropy-driven thermodynamics observed in the experimental results.
